# Commercial antibodies and their validation

**DOI:** 10.12688/f1000research.4966.2

**Published:** 2014-10-15

**Authors:** JLA Voskuil

**Affiliations:** 1 Everest Biotech Ltd, Upper Heyford, OX25 5HD, UK

## Abstract

Despite an impressive growth in the business of research antibodies a general lack of trust in commercial antibodies remains in place. A variety of issues, each one potentially causing an antibody to fail, underpin the frustrations that scientists endure. Lots of money goes to waste in buying and trying one failing antibody after the other without realizing all the pitfalls that come with the product: Antibodies can get inactivated, both the biological material and the assay itself can potentially be flawed, a single antibody featuring in many different catalogues can be deemed as a set of different products, and a bad choice of antibody type, wrong dilutions, and lack of proper validation can all jeopardize the intended experiments. Antibodies endorsed by scientific research papers do not always meet the scientist’s requirements either due to flawed specifications, or due to batch-to-batch variations. Antibodies can be found with Quality Control data obtained from previous batches that no longer represent the batch on sale. In addition, one cannot assume that every antibody is fit for every application. The best chance of success is to try an antibody that already was confirmed to perform correctly in the required platform.

## Introduction

Based on feedback from about 10 years ago, scepticism and mistrust towards commercial antibodies was already commonplace. Researchers in the academic environment preferred generating antibodies in-house by making use of the animal facilities in their faculties. At the time, the availability of commercial antibodies was not as extensive as it is today, and therefore it was unlikely that a scientist would find an antibody fitting their requirements. The present situation is quite different, yet the complaints remain. The number of commercial antibodies has escalated in the last decade, and so has demand. In contrast to 10 years ago when Western Blot (WB), ELISA and ImmunoHistoChemistry (IHC) were the most used assay types, at present antibodies are increasingly used in more sophisticated platforms such as flow cytometry, multiplex assays, immune-mass spectrometry and other capture-based assays as modern technologies have made them widely accessible. Along with this increased variety of platforms, demand for fit-for-purpose (F4P) antibodies is increasing, while disappointment by the performance of commercial antibodies remains an ever present experience.

Despite the negativity described above, the complexity of generating F4P antibodies has made the research-antibody trade one of the fastest growing markets in the life science industry. Not only has the number of traders increased, the traders also enjoyed a substantial growth in their business. There seems to be no stop in the increasing demand for commercial antibodies for research purposes. Yet, even today, the complaints of poor performance remain the biggest problem in the research antibody industry. Attempts to release multiple antibodies targeting the same protein did not make much of a difference so far. The reasons for this are outlined below.

The scientific community is struggling with the complexity that research antibodies bring to the lab, and therefore each complicating factor is discussed separately before we can build a general picture of how to benefit optimally from commercial antibodies.

## Specificity, affinity, background and noise

### Specific binding of non-specific antibodies

The term non-specificity is used when an antibody binds to unintended proteins. Each antibody molecule has a certain affinity to one part of the protein called an epitope, and this affinity is determined by the epitope’s amino acid sequence. It is therefore very difficult to find antibodies that react exclusively to one protein when this protein is very similar to other (closely related) proteins. Only antibodies that will bind to a unique epitope will react specifically to its intended target protein. However, most antibodies do not bind to unique epitopes and so they will cross-react.

In the case of shared epitopes between closely related proteins cross-reactivity is inevitable. Then the actual binding of the antibody may be specific, yet the antibody is deemed non-specific in relation to the intended target protein. Further diluting the antibody and optimizing blocking conditions will not work in these cases. In other words, the specificity of an antibody relies on the uniqueness of the protein part it binds to (i.e. the epitope).

An antibody specific to an epitope that is shared between one or two other (closely related) proteins may not be useless. It may still be useful in tissues or cell types where those cross-reacting proteins are not present. Or the scientist can take advantage in relating the intensities of bands representing the different proteins in Western blot.

Proteins unrelated to the intended target protein may have epitopes similar but not identical to the specific epitopes. Then the antibody’s affinity for the similar epitope will be lower than for the specific epitope. This will result in cross-reactivity with a proportionate lower signal, called non-specific background.

### Non-specific background

Non-specific background can be reduced by further diluting the antibody. The reason is simple: by diluting the antibody only higher affinity interactions are sustained. The lower affinity interactions (to remotely similar epitopes) will not last at lower antibody concentrations. Proper blocking conditions can also help to prevent low affinity interactions. NaCl will interfere with weak hydrostatic interactions while non-ionic detergent (for example Tween-20) will interfere with weak hydrophobic interactions between the antibodies and the unintended target proteins. Increasing the concentrations of such blocking agents may help to reduce the non-specific background.

### Noise

Poor experimental conditions will incur random noise and this is typically not related to the primary antibody. Lacking certain blocking components or the use of dirty containers/contaminated buffers are usually to blame. Especially in fluorescence-based assays, noise can be a big issue. There is a risk of antibodies being dismissed prematurely because non-specific background and noise are not considered and dealt with separately.

### Secondary antibody derived signals

In addition to primary antibody-derived issues, the secondary antibody can be a source of problems as well. The quality of the secondary antibody can be tested by side-by-side comparison of a complete experiment with another experiment lacking the primary antibody. Noise and background caused by the secondary antibody will become apparent in this negative control.

## Antibody types

The term “polyclonal antibodies” (pabs, as opposed to mabs for monoclonal antibodies) is ambiguous and can be the cause of some confusion. There are pabs raised against the full length protein, pabs raised against large protein fragments and pabs raised against small peptides. In addition, there are differences between antiserum, proteinA/G-purified, and antigen affinity purified pabs.

The general concept of pabs is that they represent a collection of antibodies raised against multiple epitopes and possibly against multiple proteins. When an antibody is raised to an entire protein, it is easy to see how multiple parts of the protein will generate a collection of different specificities and affinities. This is beneficial for certain applications such as immune precipitation (IP) and WB where cross-reactivity is easily spotted by the difference in molecular weight (unless there is cross-reactivity to proteins with the same molecular weight) compared to the intended target protein. Pabs can also be useful in IHC, as long as there is no cross-reaction with any other proteins present in the tissue sections of interest. The specificity of an antibody can be addressed by comparing endogenous expression levels to knock-down expression levels, comparing un-induced cells to induced cells (with elevated expression levels) or by looking at tissues where the location of the protein of interest is expected in one particular compartment or cell type.

Antibodies raised against a protein fragment will show higher specificity when the amino acid sequence of the fragment is unique in the proteome. Then the above mentioned advantages of pabs are combined with the uniqueness of the antigen. Although such antibodies may not compete with the mono-specific characteristics of mabs, they can work together with mabs in sandwich type ELISA and IP; using one as the capturer and the other as the reporter.

When the chosen antigen is a small peptide of the size of an epitope instead of a protein or a protein fragment, then the mono-epitopic characteristics of mabs are approached. It would be a prerequisite for this approach to have the peptide antibodies affinity purified using the antigen, thus giving high affinity pabs the upper hand over low affinity mabs. The cost of generating peptide-specific antibodies is also competitive with the cost of generating mabs because neither expensive screening and sub-cloning, nor antigen purification steps are required. A 10–15 amino acid peptide is easily and quickly synthesised and delivered by a specialist service. Just linking the peptide to a carrier protein and dialysis is required to have the material ready for immunization. And the peptide will subsequently be used for the affinity purification.

Peptide antibodies therefore are versatile tools that combine the mono-specificity attributed to the mabs with the high affinity attributed to polyclonal antibody while keeping the production costs low. In addition, the epitope (peptide sequence) for a peptide antibody is known from the product sheet, while caution is required when a mab is used without its epitope characterized and/or published. The one advantage of mabs over peptide antibodies is the mab’s longevity: As long as the hybridoma clone remains stable, the exact same antibody will be generated. This makes monoclonal antibodies preferred over pabs for commercial kits.

Antiserum and protein A/protein G purified IgG still have a mixture of affinities and specificities. From all pabs, only antigen-affinity purified antibodies will have the highest grade of specificity and affinity, and particularly so when they are peptide-derived. In my opinion peptide antibodies are ideal for research purposes, while monoclonal antibodies are ideal for long-term repeated standard assays. Yet, peptide antibodies serve as a (temporary) alternative as long as a proper mab is not available for the standard assays.

## OEM world

### Batch-to-batch variations

The vast majority of vendors do not manufacture all the antibodies on the catalogue, and most of their antibodies have been obtained from a wide variety of different manufacturers from all over the world under OEM agreement (Other External Manufacturer). Such an agreement usually has a clause to forbid the supplier from publishing which of their products are sold by their OEM vendor. The vendors keep up the appearance that they themselves are the primary source of all their antibodies. This enables them to keep QC data on the product sheet that were generated many years ago thus keeping the sales going, while the actual antibody that generated these data may have sold out and has been replaced by successive other batches (from different animals) and the current batch on sale may no longer be able to generate such data at all.

Even monoclonal antibodies suffer from batch-to-batch variations, but not to such severe extent as some types (see above) of polyclonal antibodies. Nonetheless, certain hybridoma clone numbers are still being used for decades while, just like with cell lines, hybridomas cannot be the same after so many passages anymore. It is therefore misleading to use QC data that were generated decades ago, unless the current batch has proven to still be capable of generating such data (in which case one might as well show the latest version of the data).

Assay developers are advised to buy antibodies straight from the manufacturer and ask for a free validation sample from a large batch in stock. Once validated for the required platform, the same batch then can be purchased in bulk so to prevent batch-to-batch variations during the entire project. Identifying the manufacturer can be a challenge though, and the only way to find them is to start looking for overlap between the product sheets from the different vendors. This way, a list can be generated at which point one can guess who the manufacturer was based on additional details still present on the manufacturer’s product sheet but not elsewhere.

### QC data on the vendor’s product sheet

Vendors accrue data from their own customers or from their own QC department, thus making the OEM product look unique. This way the same antibody can show different QC data on different catalogues. And while batches run out and are being replaced by others, it can happen that a vendor has still some of the old batch in stock, while another vendor will keep the QC data obtained from the former batch on their product sheet. From this moment on customers start to buy products that are no longer necessarily reflected by their product sheet.

### Same antibody on many catalogues seemingly different by their QC data

Vendors do not only obtain their antibodies from the original manufacturers. There is a network of vendors obtaining each other’s catalogue items. Consequently, the same antibody starts to occur several times in one catalogue: one time with the current QC data provided by the original manufacturer, and one or more times with QC data obtained from the other vendor’s direct customers or QC department. Potentially, assay developers buy several antibodies from several vendors thinking they are buying different antibodies, yet a number of them originate from the same manufacturer’s catalogue number.

## Reasons of performance failures

### Storage

Antibodies deteriorate by repeated freeze/thaw cycles. Such cycles should be kept to an absolute minimum. One way is to keep aliquots frozen at all times and have one aliquot in the fridge for daily use until finished. Primary antibodies usually come with preservatives to keep them good at 4°C for many months. Please be aware that some freezers are opened extremely often during a working day and then antibodies stored at the front may endure freeze/thaw cycles because of the frequent and long browsing and possibly in combination with the summer sun shining straight in. When this applies, antibodies are best kept in a different freezer that is less often opened (eg. -80°C).

### Western blot

Western blot is seen by many as the easiest and most straightforward type of immune assay. This systematic underestimation is thought to be holding back science at a great scale. With so many pitfalls unrecognized, many good antibodies are dismissed after one or two poor experiments. The Western blot is possibly the most complicated immune assay because of the many layers where the assay can fail. The most trivial problems and their remedies are highlighted in Table 1.

**Table 1.** 

Western Blot Failures	Remedies
Poor choice of tissue type or cell line	• Check vendor’s product sheet for positive control • Check literature for expressing cell types to use as positive control
Proteolysis in lysate	• Use freshly prepared protease inhibitors • Expose the lysate to reducing conditions in sample buffer shortly before gel loading
Inadequate reducing conditions	• Add fresh 2ME or DTT • Boil for 3min in 2ME, or heat to 60C for 10min in DTT
Underloading/overloading	• Check blot with Ponceau red before immunolabeling
Inadequate transfer at certain MW/air bubbles	• Check blot with Ponceau red • Adjust methanol concentration in transfer buffer
Too long incubation time	• Background reduced by shorter incubations and more stringent blocking conditions
Bad secondary antibody	• Avoid old secondary borrowed from elsewhere • Try several providers • Check species reactivity and use affinity purified with minimal x-reactivity to serum proteins of the species of interest
Bad substrate	• Use fresh substrate • For successive exposures, replace substrate timely as it expires by the conjugated enzyme

Potential problems may have already occurred during preparations of the biological material to be analyzed, well before the assay takes place. Proteolysis and oxidation are two notorious factors that will introduce low reproducibility of results when comparing different lysates from the exact same cell type with each other. The conditions (including temperature) of protein separation in SDS PAGE will influence the banding patterns. And finally, different tissue types and cell types do not necessarily give mutually identical patterns in WB. It depends on the type of protein of interest and whether any posttranslational modifications (PTM) and/or degradation may take place in a different fashion in each cell type. The PTMs may be prone to (partial) removal during the lysate preparation by endogenous enzymes. Proper inhibitors (making sure they did not get inactivated during preparation) should be added to the lysates to minimize such impairments. Hence, a commercial antibody shown to work properly in one tissue type or species should still be validated in other tissue types or species. And it is best advised to generate several lysates of the same cell type or tissue type at different days before starting analysis of all of them, side by side.

### Immunohistochemistry and immunocytochemistry (IHC/ICC)

When cells, tissue blocks or their sections have been stored after dehydration, the structure of the proteins in the cells will have changed thus affecting the binding of antibodies. Antigen/epitope retrieval will restore this, but its success is very dependent on the method of retrieval and the method may have to be adjusted from antibody to antibody (or from target protein to target protein). Heat induced epitope retrieval (HIER) can be done at pH6 and at pH9 by either high pressure steaming or by microwave. The quality of results can heavily depend on the conditions (pH value, temp, the heating duration, and the method of heating). When all attempts through HIER fail, one could opt for protease induced epitope retrieval (PIER), although some scientists prefer PIER over HIER as their sole approach.

When fresh tissues have been fixed in alcohols or acetone, one usually does not store the samples, and cryosectioning and subsequent probing with the antibodies go ahead in one flow. In this case epitope retrieval may not be required. I would however recommend epitope retrieval if the tissues have been stored long-term in the alcohol or acetone before cryosectioning. The dehydration of the tissues may mimic the paraffin embedding procedure, particularly in the case of storage in the hydrophobic acetone. Hence, rehydration of the tissue during the removal of the alcohol/acetone may still not be enough to expose the epitopes fully (again) after long term dehydrated storage.

### Immunofluorescence (IHC, ICC, FC)

Fluorescent labels are commonly used in Flow Cytometry (FC), ImmunoCytoChemistry (ICC) and in IHC as well. The major issue with fluorescence is the noise. Because of the high sensitivity of this detection type, noise is much more prevalent compared to other less sensitive detection types.

As with all assays, different dilutions of the primary (including one without primary) will have to be compared in order to appreciate the level of constant noise. One has to be careful in choosing the right blocking agents, but more importantly, one has to take into account the presence of endogenous molecules (even at low abundance) that bind to either the fluorophore itself or to its conjugated carrier (for example avoid using streptavidin-fluorophore when there is endogenous biotin, and avoid using anti-IgG-fluorophore when there are endogenous Ig-receptors).

### Immunoprecipitation (IP/ChIP)

Some antibodies will not pull their target proteins down under certain conditions. Particularly when working with linear epitope antibodies (e.g. peptide antibodies) one is advised to compare native conditions with denatured conditions (reduced and in presence of 0.1% SDS) as a positive control.

During the classic method of IP, a network of antigen and polyclonal antibodies is precipitated and analyzed. A polyclonal antibody generated to the entire protein is essential for this method as many epitopes are involved in the network formation that is precipitated. However, when the target protein is bound to one or more other proteins, the variety of epitopes for the antibody to bind to become limited and the network may not be stable enough for this classic approach. It is therefore recommended to have a control of denatured protein IP side-by-side with the actual experiment.

When using epitope-specific antibodies (monoclonal or peptide-polyclonal) the target protein (complex) is brought down by beads. This approach makes the scientist independent on an antibody-antigen network to form. When the right epitope-specific antibodies are used, extra information can be generated about the binding sites for the interacting other proteins.

### Microwell platforms

This assay type represents any micro-well formatted immunoassay. They all have in common that one reagent is coated to a stationary phase (usually the bottom of the well), and other reagents react with the coated reagent proportionally to the content of the analysed material within a natural matrix. Matrix could be a body fluid (plasma, serum, urine, etc), a culture supernatant, or a buffered solution spiked with biological material from which one constituent needs quantifying.

The biggest hurdle in such assays is the notorious matrix effect. To put it simply: the matrix contains molecules that interfere with the reaction between the antibodies and the antigen to be quantified. This interference can be visualized by making serial dilutions of the matrix. When the matrix is diluted with a factor two and the measured antigen (present at sub-saturating levels) therein does not read a reduction by a factor two along the way, you have established matrix effect. Also when readings do not correlate to the levels of antigen spiked into the matrix of interest, there will be matrix effects. Matrix effects are best dealt with by diluting the matrix in assay buffer to such extent that the antigen can be quantified without interference from the matrix. It is essential to establish matrix effect every time a new assay is set up. When a new antibody is introduced in an already existing assay, the matrix effect may respond differently from the previous antibody. A calibration curve needs to be made in the same matrix and run in parallel with each assay.

Polyclonal antibodies may perform better after being pre-adsorbed to abundant serum proteins as a blocking step. This should be a compulsory step when using the antibody in matrix containing high levels of serum protein, especially when all proteins in the matrix were labelled before the antibody was added. One needs to be aware that most commercial primary and secondary antibodies have not been pre-adsorbed to serum proteins. Some detergents may also minimize matrix effect.

### Reproducibility

One should not jump to conclusions after a single experiment. Even positive data may sometimes be false positive and lack of signals may be due to trivial factors that can be solved by systematic trouble shooting. Therefore conclusions can only be drawn once identical results have been obtained by the same experiment carried out at different times.

### Transportation-related impairments

Anyone working in a subtropical climate (eg. southern state of the USA) knows how hot it becomes on a sun baked parking space during summer and how temperatures can soar inside a delivery van parked out there while taking care of a delivery. Antibodies are inherently made to work at 37–42°C, but it remains a protein and it can cook until inactive when temperatures go well over 50°C. Every antibody is different from the next and the majority will survive the above mentioned conditions. When antibodies in solution arrive on frozen icepack, one can assume that the integrity of the antibody has been maintained during transport. Lyophilized antibodies are more resistant. It is the user’s responsibility to ensure that the ordered antibody is received in proper packaging (particularly during hot weather conditions) and complain to the vendor when this is not the case. When an antibody does not perform as expected based on the product sheet and the antibody was delivered under hot conditions, it is time to ask for replacement.

### Endorsement by publication

Today’s advanced internet facilities enable vendors to search for publications describing successful use of their antibodies. However, some publications mistakenly attribute an antibody to the wrong vendor. And sometimes an antibody is correctly reported but no data generated by it are to be seen or presented in the paper, nor in the supporting documentations. In such cases the internet search delivers false-positives, and examples can be found in each vendor’s catalogue. Many such papers are inaccessible to the vendors and therefore they cannot always double check themselves. Although this is a relatively rare phenomenon, it is real all the same and therefore the customer is advised to double check the contents of the referenced paper when such endorsement is a requirement for the scientist to purchase this antibody. Each vendor will appreciate the feedback when a customer identifies a false-positive reference, and we urge customers to contact the vendor when such reference has been identified so they can remove it from the product data sheet.

Although there is a recent change in trend, the vast majority of publishers still do not demand to specify the used antibodies by their catalogue number in scientific papers. Since most catalogues have more than one antibody to one protein, a mere description of an antibody from vendor X to protein Y is not sufficient and prevents peers from replicating the described experiments.

## General principles

Every researcher in the lab will have different wishes and demands on how an antibody should perform. Ideally, an antibody meets all demands one can think of. Unfortunately this does not always happen. Each antibody has its own unique characteristics. It may work very well in one or two types of assays (for example in WB and IHC), but not in other platforms. If one needs an antibody for quantification in micro-well format, one should not test in WB or IHC. Many WB antibodies do not work in IHC and many IHC antibodies do not work in WB. Yet, all combinations of the above are feasible, and to certain target proteins all antibodies work in all applications tested.

Sadly, there are also many proteins out there that refuse to generate antibodies fit for any application. Hence, the choice of target protein is a big factor determining the versatility of antibodies or whether it is fit for purpose at all. The host species can also be a factor. It is commonly known that when mammals fail to produce a decent antibody one has to generate bird antibodies (chicken).

Validation can also be restricted by a regulatory environment. Laboratories regulated by Good Laboratory Practice (GLP) will have to follow fixed procedures for antibody validation (see http://www.mhra.gov.uk/Howweregulate/Medicines/Inspectionandstandards/GoodLaboratoryPractice/Structure/ and http://www.epa.gov/compliance/monitoring/programs/fifra/glpsops.html). In this case, the quality control data on the product sheet are hardly relevant. The operator in the GLP lab will have to follow the obligatory procedures from the start anyway. Here, one has to decide on purchasing the antibody with the highest chances of success. Here the decision can go wrong when one picks an antibody with superior QC data that is from irrelevant applications. One tends to think that an antibody without QC data is less likely to be successful for the required assay in the GLP lab than an antibody with nice WB and/or IHC data. It is a difficult choice to make as each purchase has to undergo this obligatory and therefore expensive validation procedure. The best choice is an antibody that already has proven itself in the relevant assay type. It is therefore recommended to first try a panel of different antibodies (from different manufacturers) in a much cheaper feasibility study before making that choice. It is worth asking manufacturers for antibodies that cannot be found on any catalogue. Many antibodies are waiting to be tested in assays not accessible to the manufacturers, and so they are not for sale (not working in WB or IHC) and a free sample is often made available if feedback on the results is promised in return.

## How to validate an antibody

An antibody is meant to bind to the protein intended. This confirmation is a minimal requirement for the product’s datasheet. One should never purchase a product without this confirmation on the product sheet. Usually this confirmation is established by direct ELISA with a titre. Alternatively, a recombinant protein or purified protein (or fragment thereof) may be stained in WB. This is not yet a validation of any kind! It merely confirms that the antibody has a certain affinity to the intended target protein.

Validation starts with comparing the antibody’s affinity to the intended target protein with its affinity to all other proteins occurring in the natural environment of the intended target protein. In other words, the antibody needs to be able to specifically bind to its intended target while it does not bind to the vast majority of all other molecules that naturally surrounds the target. For this reason it is good practice to compare the binding of the antibody on two identical mixtures of proteins, one with the target and one without the target. This may translate into comparing matrix, lysate or tissue containing endogenous target levels with matrix, lysate or tissue containing knocked-down or knocked-out target levels. Or as an alternative, matrix, lysate or tissue with low target levels compared to matrix, lysate or tissue with artificially increased target levels.

More often than one would wish, the results of the above mentioned tests are not going to be black and white. When successful, a clear preference is observed to the intended target, but with a certain level of background. At this stage one has to optimize conditions so to increase the signal/background to acceptable levels. One has to take into account that when the antibody is binding to common epitopes, this antibody is going to be cross-reactive with related proteins sharing these epitopes when also present in the mix. This cross-reactivity then invalidates the antibody and a better antibody needs to be identified. When the antibody is mono-specific to one defined and unique epitope, one has to take into account that such an antibody will still bind to similar epitopes albeit at lower affinity. The background occurring from proteins with such epitopes can be reduced by further diluting the antibody and by reducing the primary incubation time. Consequently negative control tissues or cells still show signals when the antibody was used at too high concentration. Please note that this principle is prone to be overlooked by the world of antibody therapeutics during early clinical trials, thus posing the risk that a potential therapeutic antibody gets dismissed when it will bind to other proteins at high dose with potential dire consequences!

Background can also derive from added reagents required for signal reporting. A bad secondary antibody can be identified by comparing the complete assay with the same assay but without the primary antibody. Finally, random noise is most likely produced by the reporting chemicals when the blocking conditions and or buffer constituents have not been optimal.

In WB, background can be seen as extra bands, while noise can be seen as random spots. In IHC, background will still stain certain structures in the cell (albeit different from the expected structures), while noise will show stains overlapping different cellular structures, thus showing lack of specificity of the stain itself. In fluorescence, noise will become apparent as a constant when different dilutions of the primary are compared.

The complexity of commercial antibodies is for a great deal owed by the OEM agreements. Everyone should be aware that the same antibody appears multi-fold on many catalogues worldwide and not always with identical QC data. Another complication is the formulation and antibody type on offer. A monoclonal antibody can be offered as a purified IgG in known mg/ml concentration, while another catalogue offers the same monoclonal antibody in culture media without any specifications. A polyclonal antibody can be offered as affinity purified by one catalogue and as antiserum by another. Scientists should be aware that when the (exact) epitope is not given or known, the antibody needs a more robust validation study than when the epitope is specified.

The many layers of complexity outlined above give rise to combinations of problems that are challenging to solve. Only systematically going through each layer separately, one can finally validate an antibody without running the risk of dismissing a precious product.

## Concluding remarks

Recently, new initiatives are being developed to aid the scientists with their validation efforts; F1000Research is launching a permanent article collection to provide a platform for scientists to publish their antibody validation studies. In addition, publishers are starting to ask the authors to report the catalogue number of each antibody used in their publications. Also, last February the Resource Identification Initiative was launched ( http://scicrunch.com/resources) which provides permanent identifiers for lab resources such as antibodies, model organisms and software tools and aims to make these tools universally identifiable. These are significant steps towards our goal of increasing the confidence the scientific community has in commercial antibodies.

