# Supplementary material for: Commercial antibodies and their validation
Source: F1000Res. 2014 Oct 15;3:232. Originally published 2014 Oct 2. [Version 2] doi: 10.12688/f1000research.4966.2 (PMC4197739; doi:10.12688/f1000research.4966.2)
Supplement: Peer review history

# Peer review: Referee response for version 1

**DOI:** 10.5256/f1000research.5303.r6302

**Published:** 2014-10-10

**Authors:** Andrew Chalmers

**Affiliations:** 1 Department of Biology and Biochemistry, University of Bath, Bath, UK

This opinion article gives Dr Voskuil’s view on commercial antibodies and how researchers can make best use of them and potentially avoid a lot of frustration at the bench. He bases his opinions on his extensive experience of commercial antibodies gained during his time as Chief Scientific Officer of Everest Biotech.

The article is wide ranging and gives many useful pieces of advice that I hope will help researchers and prompt some interesting discussion. I was pleased to see the fact that many suppliers will offer the same antibody (OEM) and the fact that properly citing antibodies is important, were both covered.

One area that could have been covered in more detail is that the three dimensional structure of the epitope is important, both when the antibody is raised and in the applications that are being carried out, for example denaturing conditions in western blotting and native folding in IPs. However, no article can cover everything and this one is already wide ranging, so I don’t think it is an essential correction.

There were a few small issues that could be corrected/changed if a revised version is produced.

1. I would have thought tween-20 should be referred to as a detergent rather than non-polar?
2. In table 1, “reduce the lysate” this could be clearer, does it mean add the reducing agent just before loading or reducing the amount of lysate?
3. page 5, “to put it simple” should read “to put it simply”
4. Given this is an opinion article, it might be good to have a box summarizing Dr Voskuil’s experience (which is extensive) to explain to readers why he is qualified to provide the commentary.  

I have read this submission. I believe that I have an appropriate level of expertise to confirm that it is of an acceptable scientific standard.

# Response

**Published:** 2014-10-10

**Authors:** Jan Voskuil

**Affiliations:** Everest Biotech Ltd, UK

Many thanks for your kind and helpful comments. I will certainly look at submitting a version 2. I concur that a summary of my experience may add value, and will consider adding this to the revised article. In the meantime my experience can also be found in my LinkedIn profile.

Your recommendations are highly appreciated. However, Tween-20 is a non-ionic detergent, but not all detergents are non-ionic: SDS and urea for example are ionic detergents (also known as chaotropic agents).

Sorry that I have left out the three-dimensional epitopes. I did it on purpose since I think there is another opinion paper to be written on this very (in my opinion yet unsettled) subject. Besides, it does not add to the objective of this paper: Giving the reader insight into the pitfalls of commercial antibodies and how to avoid wasting unnecessary resources.

# Peer review: Referee response for version 1

**DOI:** 10.5256/f1000research.5303.r6304

**Published:** 2014-10-10

**Authors:** Simon Glerup

**Affiliations:** 1 Department of Biomedicine, Aarhus University, Aarhus, Denmark

Apart from research funding, difficulties in finding good research antibodies is probably one of most limiting and disabling factors in life science research.

The opinion article by Dr. Voskuil contributes with important insight into the research antibody industry. In particular, I think that most scientists are unaware of the reselling and relabeling of commercial antibodies that apparently is common practice in the industry. The article also meticulously puts forward a set of guidelines for validating the use of research antibodies, which are of broad interest to the scientific community.

It was a pleasure to read and I recommend it for indexation.

I have read this submission. I believe that I have an appropriate level of expertise to confirm that it is of an acceptable scientific standard.

# Peer review: Referee response for version 1

**DOI:** 10.5256/f1000research.5303.r6362

**Published:** 2014-10-07

**Authors:** Fridtjof Lund-Johansen

**Affiliations:** 1 K.G. Jebsen Center for Cancer Immunotherapy, University of Oslo, Oslo, Norway

What does the antibody manufacturer say?

We all know what scientists say about commercial antibodies, but what does the antibody manufacturer say? In this opinion article the CEO of an antibody manufacturing company points out that many problems may be solved if the customers pay attention to common pitfalls in antibody applications and understand how the reagents are manufactured and marketed.

The author begins with a general description of terms such as specificity, cross-reactivity, and background and continues with explaining differences between monoclonal and polyclonal antibodies. Even experienced users need to be reminded that polyclonal antibodies do not necessarily detect multiple epitopes, that there is extensive lot to lot variation in performance and that purified IgG is not the same as affinity purified antibody.

Those who use assays such as western blotting, immuno-histochemistry and ELISA on a regular basis will most likely recognize the pitfalls that are described. Experts can surely produce far longer and more detailed lists with suggestions for troubleshooting. However, for the much larger number who use these assays only now and then, the article should serve as a useful reference.

The author flags that he is CEO of a manufacturer that specializes in the production of anti-peptide polyclonal antibodies. Thus, one may expect that he has strong opinions about the relative performance of antibodies raised against peptides or full length proteins, respectively. One may of course agree that an antibody that has been raised and affinity purified against a peptide can be said to recognize a single epitope. Yet, there is no reference to any studies where anti-peptide antibodies  have been shown to be generally more specific than reagents raised against full length proteins.

For the more experienced antibody users, the open and honest description of the antibody industry is likely to be the most interesting part of the article. Most users are probably not aware of the extensive practice of buying and selling products that occurs between suppliers. It is also very useful to be warned that the validation data in product specification sheets are often not generated by the seller of the reagent and that they may not be representative of the particular lot that is sold at any given time point. The fact that the same reagent is sold under different names and with different validation data is another very interesting detail.

This is an opinion article, and should be read as such. This reviewer tends to disagree at several points. Yet, hearing what the antibody manufacturer says is well invested time.

I would recommend the following changes:

 

1. The matrix effect: page 5 right, first paragraph. The sentence "When the matrix is diluted with a factor two and the measured antigen does not read a reduction by a factor two along the way...... " may be misinterpreted. If the sample is diluted and the signal remains the same, the antigen may be saturating.
2. I was not aware that antibodies may perform better in ELISA for low abundance serum proteins if they are adsorbed against human serum proteins first. I do not understand why this would be the case, and a reference would be good.
3. I would remove the discussion about therapeutic antibodies on page 6. Therapeutic antibodies undergo testing far beyond that of antibodies used for research purposes.
4. I would not call extra bands in a WB "background" but rather cross-reactivity.

I have read this submission. I believe that I have an appropriate level of expertise to confirm that it is of an acceptable scientific standard.

# Response

**Published:** 2014-10-07

**Authors:** Jan Voskuil

**Affiliations:** Everest Biotech Ltd, UK

Many thanks for your well formulated feedback. I will systematically respond to the points you are raising:

The paper is not meant to only give an idea what the scientist in the lab should do. It should be clear that certain responsibilities also lie with the vendors. This paper should not be split into two sections: one to tell the users what to do and one to tell the users how the industry works. This paper is meant to give an unbiased overview of how trust in commercial antibodies can be gained from the market. In my opinion an integrative approach and total transparency can achieve this.

I am not in agreement that this paper is merely fit to advice scientists who only now and then uses a particular assay and who is in need of technical support. Based on a decade of feedback from both industry and academics I can assure you that all experts need some reminding from time to time. I am convinced that my explanation on how peptide antibodies differ from other polyclonal antibodies will be well received.

I hope I am convincing enough to make a case for antibodies raised and affinity purified using a short peptide approaches the mono-specificity of monoclonal antibodies, while antibodies generated to larger protein fragments or to entire proteins would per definition contain a mixture of antibodies to many different part of the antigen. This logic does not require evidence by reference.

By the way, I am CSO, not CEO of the company. I bear responsibility for the quality and scientific content on the catalog and web pages, while the CEO (another person in this case) bears end-responsibility for the entire enterprise.

I appreciate your recommendations. Once all reviews are in, I will pay a little more attention to the passages that you mention. I do have to maintain though, that extra bands in WB are considered background when you can fade them out upon further dilution of the primary antibody, while this is not the case with cross-reaction (to proteins with identical epitopes). This matter highlights that even experts sometimes need a reminder.
